# Supplementary material for: The genome sequence of the Winter Shade, Tortricodes alternella [Denis & Schiffermüller], 1775
Source: Wellcome Open Res. 2024 May 8;9:236. [Version 1] doi: 10.12688/wellcomeopenres.21494.1 (PMC11187527; doi:10.12688/wellcomeopenres.21494.1)
Supplement: Peer review history

# Reviewer report: Reviewer response for version 1

**DOI:** 10.21956/wellcomeopenres.23764.r85991

**Published:** 2024-06-19

**Authors:** Jovana M. Jasso-Martínez

**Affiliations:** 1 Universidad Nacional Autonoma de Mexico, Mexico City, Mexico City, Mexico

I reviewed the manuscript "The genome sequence of the Winter Shade, *Tortricodes alternella" .* Authors explain methods well and also provide information to find full protocols, which allow replication of the study.

 This is the first contribution of a complete genome for the genus *Tortricodes.*

 Authors mentioned that the mitogenome assembly is within the multifasta file of the genome submission; however, i recommend to upload it separately with annotation so it can be retrieved in NCBI when blasting. 

 Also, if possible, I recommend providing better-quality imaging of specimens used in the study.

Are sufficient details of methods and materials provided to allow replication by others?

Yes

Is the rationale for creating the dataset(s) clearly described?

Yes

Are the datasets clearly presented in a useable and accessible format?

Yes

Are the protocols appropriate and is the work technically sound?

Yes

Reviewer Expertise:

entomolgy, genomics, phylogenomics, systematics

I confirm that I have read this submission and believe that I have an appropriate level of expertise to confirm that it is of an acceptable scientific standard.

# Reviewer report: Reviewer response for version 1

**DOI:** 10.21956/wellcomeopenres.23764.r85990

**Published:** 2024-06-19

**Authors:** Rudolf Meier

**Affiliations:** 1 Humboldt University, Berlin, Germany

The manuscript describes the draft genome sequence of *Tortricodes alternella.* I have few suggestions for improvement:

 (1) The Abstract includes "(the Winter Shade; Arthropoda; None; Lepidoptera; Tortricidae)." Presumably, there is something wrong with the "None" part.

 (2) The following sentence under Background should be split: “It has a wingspan of 19–23 mm and narrow fore wings for a tortrix which are a variably marked in shades of grey/yellow brown with a darker cross-band, whilst the hind wings are pale brown with darker veins.”

 (3) Rephrase: "It is one of the earliest tortricids on the wing, between late January and April, and will come to light" given that the report is also for genome biologists.

 (4) You reference the "GBIF Secretariat, 2024" instead of providing the species link in GBIF. 

 (5) The species have several DNA barcodes in NCBI. I would recommend that the statement about species identification by Liam Crowley is complemented with a statement that the COI sequences also match the DNA barcodes for the species followed by the relevant access numbers.

Are sufficient details of methods and materials provided to allow replication by others?

Yes

Is the rationale for creating the dataset(s) clearly described?

Yes

Are the datasets clearly presented in a useable and accessible format?

Yes

Are the protocols appropriate and is the work technically sound?

Yes

Reviewer Expertise:

entomology, species discovery

I confirm that I have read this submission and believe that I have an appropriate level of expertise to confirm that it is of an acceptable scientific standard.
